# HLA-G UTR Haplotype Conservation in the Malian Population: Association with Soluble HLA-G

**DOI:** 10.1371/journal.pone.0082517

**Published:** 2013-12-23

**Authors:** Federico Carlini, Karim Traore, Nissem Cherouat, Pierre Roubertoux, Stéphane Buhler, Martì Cortey, Sophie Simon, Ogobara Doumbo, Jacques Chiaroni, Christophe Picard, Julie Di Cristofaro

**Affiliations:** 1 Aix-Marseille Université, CNRS, EFS, ADES UMR 7268, Marseille, France; 2 Malaria Research and Training Center, Department of Epidemiology of Parasitic Diseases, Faculty of Medicine, Pharmacy and Dentistry, Bamako, Mali; 3 Immuno-genetics laboratory, Etablissement Français du Sang Alpes Méditerranée, Marseille, France; 4 Inserm U491, Génétique Médicale et Développement, Aix-Marseille Université, Faculté de Médecine, Marseille, France; 5 Laboratory of Anthropology, Genetics and Peopling history (AGP), Department of Genetics and Evolution – Anthropology Unit, University of Geneva, Geneva, Switzerland; University of Florence, Italy

## Abstract

The HLA-G molecule plays an important role in immunomodulation. In a previous study carried out on a southern French population our team showed that HLA-G haplotypes, defined by SNPs in the coding region and specific SNPs located in 5′URR and 3′UTR regulatory regions, are associated with differential soluble HLA-G expression (sHLA-G). Furthermore, the structure of these HLA-G haplotypes appears to be conserved in geographically distant populations.

The aim of our study is to confirm these expectations in a sub-Saharan African population and to explore additional factors, such as HLA-A alleles, that might influence sHLA-G expression.

DNA and plasma samples were collected from 229 Malians; HLA-G and HLA-A genotyping were respectively performed by the Snap Shot® method and by Luminex™ technology. sHLA-G dosage was performed using an ELISA kit. HLA-G and HLA-A allelic and haplotypic frequencies were estimated using an EM algorithm from the Gene[Rate] program. Associations between genetic and non genetic parameters with sHLA-G were performed using a non-parametric test with GRAPH PAD Prism 5.

Our results reveal a good conservation of the HLA-G UTR haplotype structure in populations with different origins and demographic histories. These UTR haplotypes appear to be involved in different sHLA-G expression patterns. Specifically, the UTR-2 haplotype was associated with low sHLA-G levels, displaying a dominant negative effect. Furthermore, an allelic effect of both HLA-G and HLA-A, as well as non genetic parameters, such as age and gender possibly linked to osteogenesis and sexual hormones, also seem to be involved in the modulation of sHLA-G.

These data suggest that further investigation in larger cohorts and in populations from various ethnical backgrounds is necessary not only to detect new functional polymorphism in HLA-G regulatory regions, but also to reveal the extent of biological phenomena that influence sHLA-G secretion and this might therefore have an impact on transplantation practice.

## Introduction

### Background

The role of the non-classical class Ib Human Leukocyte Antigen-G (HLA-G) in immune-tolerance has been well documented [1], [2], [3]. Tolerogenic properties of HLA-G were initially identified in the cytotrophoblast and correlated with feto-maternal tolerance [4], [5], [6], [7]. Modulation of HLA-G expression is observed in numerous pathological situations such as tumours, viral infections, inflammatory and autoimmune diseases [8], [9], [10], [11], [12], [13], [14], [15], [16]. HLA-G immune modulatory properties seem to be important in graft acceptance, i.e. HLA-G inhibits immune effectors and protects transplanted organs from rejection [17], [18]. Several studies have shown a clinical correlation between expression of soluble and/or membrane-bound HLA-G and reduction of rejection risk in heart, lung, liver and kidney transplant patients or Graft versus Host disease [19], [20], [21], [22].

Contrary to the classical HLA class I loci, HLA-G is characterized by a low polymorphism in the coding regions. To this day, 50 HLA-G alleles have been identified, which encode 16 trans-membrane proteins (HLA-G*01:01 to G*01:04, G*01:06 to G*01:12 and G*01:14 to G*01:18) and two truncated proteins (HLAG*01:05N and G*01:13N) [23]. However, a higher degree of polymorphism has been observed in the non-coding regions 5′URR (Upstream Regulatory Region) and 3′UTR (UnTranslated Region) [24].

Several studies have suggested an association between soluble (s)HLA-G expression and specific HLA-G alleles or SNPs in the non-coding regions. Notably, HLA-G*01:04 and G*01:05N have been respectively associated with high and low HLA-G secretion [25], [26]. Among the 29 SNPs identified in the HLA-G 5′URR, some are located within or near regulatory elements and seem to affect regulatory binding factor affinity. In particular, the −725, −716, −201 and −56 positions have been independently associated with HLA-G expression [27], [28], [29], [30]. In the 3′UTR, four polymorphisms appear to be implicated in the regulation of HLA-G expression levels. The +3142 position affects the affinity of specific microRNAs (miRNA) for HLA-G mRNA. The +3187 and +3196 positions, located near an AU-rich motif in the HLA-G mRNA, have been associated with its stability. The exon 8 14-bp *insertion/deletion* polymorphism has been associated with differential sHLA-G expression (i.e. the *ins/ins* genotype displays a lower level of sHLA-G than the *ins/del* and *del/del* genotypes) [31],[32],[33].

HLA-A is the closest functional gene to HLA-G. The genetic distance between these two genes is approximately 150 Kb [34]. Several studies have reported medium to high levels of linkage disequilibrium (LD) between different HLA-A and HLA-G alleles [35], [36]. Numerous non-functional genes (pseudogenes) such as HLA-H can be found between these two genes. Two HLA-A allele groups, HLA-A*23 and HLA-A*24 were previously reported to be associated with a large-scale deletion of 50 kb including the HLA-H pseudogene in the region that precedes HLA-G [37], [38], [39], [40]. The LD between HLA-A and HLA-G alleles may be due to the relatively short genetic distance (and limited recombination events) between them, but may also be the reflection of some, yet unknown, biological constraint.

Castelli et al. defined 8 UTR HLA-G haplotype groups using sequenced SNPs in the 5′URR, 3′UTR and coding regions in a Brazilian population [24]. This low variability in such an admixed population suggests that a stabilizing selective effect acts on UTR haplotypes, possibly involving sHLA-G expression patterns. When focusing on regulatory regions, these authors found a balanced effect using Tajima's D and Fu and Li's F neutrality tests.

Based on this study, our team investigated HLA-G UTR haplotype conservation and its association with the expression of sHLA-G in serum from Volunteer Bone Marrow Donors (VBMD) from South-eastern France [41]. Our study focused on UTR haplotypes defined by four SNPs in the 5′URR region (−725 (C/G or T), −716 (G/T); −201 (G/A) and −56 (C/T)), four SNPs in the 3′UTR (*ins/del* exon 8, 3142 (C/G), 3187 (G/A) and 3196 (C/G)) and coding HLA-G alleles defined by eight SNPs. In this preliminary study we confirmed the conservation of the HLA-G UTR haplotype structure and its allelic association by identifying the eight previously defined UTR haplotypes [24]; Importantly, this preliminary study suggested a correlation between UTR haplotypes and sHLA-G expression. Indeed, two UTR (UTR-5 and UTR-1) were correlated with high sHLA-G secretion, whereas one (UTR-2) was correlated to low sHLA-G secretion. Finally, this preliminary study did not confirm the effect of the *ins/ins* genotype, consensually associated with lower sHLA-G expression: the *ins* allele was present in both UTR-5 and UTR-2, which we showed to be respectively associated with high and low sHLA-G secretion. However, another team has contradicted these preliminary results, notably showing that UTR-2 and UTR-5 are respectively correlated with intermediate and low sHLA-G levels (personal communication).

### Hypothesis and objectives

On this basis, we propose to investigate the following hypotheses: (1) the restricted number of UTR HLA-G haplotypes and their structure may reflect selective forces associated with differential expression of sHLA-G and its biological significance. This supposition should be confirmed in a sub-Saharan African population since these populations generally display higher genetic diversity and lower levels of linkage disequilibrium compared to populations from other continents. (2) Accordingly, the association between specific UTR haplotypes and sHLA-G levels should be reproducible at plasma level in Malian samples. (3) Finally, the HLA-A gene, due to its proximity to the HLA-G gene, may influence the expression of sHLA-G. Thus, haplotype conservation might be extended to the HLA-A gene.

## Materials and Methods

### Subjects

Sample collection was conducted by the *Malaria Research and Training Center, Department of Epidemiology of Parasitic Diseases, Faculty of Medicine, Pharmacy and Dentistry, Bamako, Mali*. Participants provide their written informed consent. Kin provide their written informed consent on the behalf of the children participants involved in this study. The study protocol and the consent procedure were approved by the *Ministère de l'Enseignement Supérieur et de la Recherche* in France and by the *Comité d'éthique institutionnel de la faculté de médecine, de Pharmacie et d'odontostomatologie* in Mali.

Blood and plasma samples were collected from 229 unrelated Malians after informed consent, in the villages of: Bandiagara (n = 61), Binedama (n = 12) Madougou (n = 53), Manteourou (n = 51), N'Gono (n = 21) and Petaka (n = 31). The ethnic groups were: Dogon (n = 152, linguistic group Niger-Congo-Atlantic), Peulh (n = 68, linguistic group Niger-Congo-Atlantic), Tamashek (n = 5, linguistic group Afro-Asiatic-Berber) and Bambara (n = 4, linguistic group Niger-Congo-Mande). This cohort was composed of men and women (respectively n = 125 and n = 105, *sex ratio* of 1.2). The mean age was 47.7 years old (range: 26–88 years) for adults and 9.7 years old (range: 3–25 years) for children.

DNA was extracted in Mali from a 200-µl whole blood sample using the QIAmp Blood DNA kit (Qiagen, Courtaboeuf, France) according to the manufacturer's instructions. Genomic analyses and serology were performed in Marseilles respectively on genomic DNA and plasma from the same cohort. 229 individuals were successfully analyzed for HLA-G coding alleles, 5′URR and 3′UTR polymorphisms and 195 individuals were analyzed for HLA-A coding alleles. sHLA-G level was determined in plasma samples from 219 individuals.

### HLA-G 5′URR, 3′UTR and alleles genotyping

A homemade primer extension method was used to simultaneously analyse 16 SNP scattered along the HLA-G gene: 8 SNPs (codons 13C/T rs17875397; 31 A/T rs41551813; 54 A/G rs41545515; 110 C/A rs12722477; 130 C/T rs41557518; 159 T/C rs55916353; 219 C/T rs45530733 and 258 C/T rs12722482) defining HLA-G alleles (HLA-G*01:01 to G*01:09), 4 SNPs in the 5′ URR region (−725 C/G/T rs1233334, −716 G/T rs2249863, −201 G/A rs1233333 and −56 C/T rs17875397) and 4 SNPs in the 3′UTR region (*ins*/*del* exon 8 rs66554220; 3142 C/G rs1063320; 3187 G/A rs9380142 and 3196 C/G rs1610696) [41]. HLA-G genotypes were analyzed using GeneMapper® 4.0 with specific detection parameters. This genotype identification method does not allow the detection of alleles G*01:10 to G*01:18.

### HLA-A genotyping

Luminex™ technology (HLA-A-One Lambda LABType® SSO) was used to determine HLA-A alleles at an intermediate resolution using the manufacturer's kit. The HLA-A allelic assignment is based on the HLA sequences listed in the official IMGT/HLA database 3.12.0 May 2013 [23].

### Soluble HLA-G protein dosage

Measurement of soluble isoforms HLA-G1 and -G5 was performed *in duplicate* on plasma samples using the ELISA test (Biovendor®, Prague, Czech Republic) according to the manufacturer's protocol. For each ELISA test, 6 samples were used as controls, 2 with low levels, 2 with median levels and 2 with high levels, to evaluate the repeatability and reproducibility. Inter-plate sHLA-G value variability was normalized using the mean value of a control plate.

### Statistical analyses

HLA-G genotypes were automatically converted from output files (.txt) exported from GeneMapper 4.0 into coding alleles and UTR using an in-house computer program, readable by the ‘Phenotype’ application of the Gene[Rate] computer tool package (http://geneva.unige.ch/generate) [42].

Significant deviations from expected values at Hardy Weinberg Equilibrium (HWE) for all the 16 HLA-G SNPs were tested using a nested likelihood model [41].

Frequencies for HLA-G alleles, SNPs in the 5′ and 3′ regions, HLA-A alleles, UTR∼HLA-G and HLA-A∼HLA-G∼UTR haplotypes were estimated using an EM algorithm from the Gene[Rate] program [42] and confirmed using the EM and ELB algorithms from the Arlequin v3.5.1.2 package [43].

Two-locus linkage disequilibrium (LD) was tested for the 16 HLA-G SNPs by a conventional goodness-of-fit test with the Arlequin v3.5.1.2 package [43]. LD for HLA-A and HLA-G alleles and for HLA-A alleles and HLA-G UTR were tested by two methods: a likelihood-ratio test on the frequency estimations, comparing the joint (haplotypic) estimation for both loci with the product of the individual (allelic) estimations for each locus, all estimations being made under the assumption of HWE (significant when p<0.05); and a parametric re-sampling test of the allele frequencies (significant when quantile >950).

Gametic association between specific pairs of alleles is provided as a list of standardized residuals for each observed haplotype. The null hypothesis of independence of the loci implies a gaussian distribution of deviations and, by convention, absolute values over 2 are considered to be significant. In order to establish the relationships among the HLA-A and HLA-G haplotypes, a Median Joining (MJ) network [44] based on protein sequences was constructed using the program Network (www.fluxus-engineering.com/network). The HLA-A alleles were grouped into five lineages previously defined [45], [46], [47].

Associations between sHLA-G and genetic polymorphism (SNPs, allele or haplotype) or non genetic parameters (sex) were tested with non-parametric tests performed with GRAPH PAD Prism 5. Mann-Whitney t-test was used to test two modalities. Kruskal-Wallis one-way ANOVA followed by Dunn post-hoc test was used when there were more than two modalities. Statistical correlation between age and sHLA-G levels was tested using Spearman's rank test.

## Results

### HLA-G and HLA-A Genotyping

#### 5′URR, 3′UTR SNPs and HLA-G allele frequencies

Frequencies of the HLA-G alleles, the 4 SNPs in the 5′URR (−725 C/G/T, −716 G/T, −201 G/A and −56 C/T) and the 4 SNPs in the 3′UTR (*ins/del*, 3142 C/G, 3187 G/A and 3196 C/G) in the Malian samples are shown in Table 1. These results are compared with those previously reported in French Volunteer Bone Marrow Donors (VBMD) [41].

**Table 1:** Frequencies (Fq) and absolute value (N) of coding HLA-G alleles, 5′URR and 3′UTR SNPs in the Malian population.

HLA-G alleles, 5′URR and 3′UTR SNPs	*Fq Malian*	*N Malian*	*Fq VBMD * [41]	*N VBMD*	*p* value
G*01:01	*0,452*	*104*	*0.795*	*196*	***
G*01:04	*0,262*	*60*	*0.123*	*30*	***
G*01:03	*0,157*	*36*	*0.028*	*7*	***
G*01:06	*0,022*	*5*	*0.033*	*8*	NS
G*01:05N	*0,105*	*24*	*0.021*	*5*	***
−56 C	*0,84*	*192*	*0.972*	*239*	***
−56 T	*0,16*	*37*	*0.028*	*7*	***
−201 G	*0,451*	*103*	*0.598*	*147*	***
−201 A	*0,549*	*126*	*0.403*	*99*	***
−716 T	*0,549*	*126*	*0.594*	*146*	***
−716 G	*0,451*	*103*	*0.407*	*100*	***
−725 C	*0,79*	*181*	*0.816*	*201*	NS
−725 G	*0,058*	*13*	*0.156*	*38*	***
−725 T	*0,152*	*35*	*0.028*	*7*	***
ex8 del	*0,441*	*101*	*0.693*	*170*	***
ex8 ins	*0,559*	*128*	*0.308*	*76*	***
3142 C	*0,291*	*67*	*0.569*	*140*	***
3142 G	*0,709*	*162*	*0.431*	*106*	***
3187 A	*0,879*	*201*	*0.661*	*163*	***
3187 G	*0,121*	*28*	*0.339*	*83*	***
3196 C	*0,719*	*165*	*0.794*	*195*	*
3196 G	*0,281*	*64*	*0.206*	*51*	*

Results are compared with previously published results on VBMD [41]. Statistical differences between the two populations frequencies were measured using a chi-squared test (NS: not significant; *: p<0.05; **: p<0.01; ***: p<0.001).

No significant differences were observed for allele frequencies between villages or between ethnic groups, the Malian data were thus pooled together for the analysis.

Five alleles were found in the Malians: G*01:01 (45.2%)>G*01:04 (26.2%)>G*01:03 (15.2%)>G*01:05N (10.5%)>G*01:06 (2.2%). The five HLA-G alleles previously reported in the French population (VBMD) were all observed in Malians. Malian samples displayed a significantly higher frequency (p<0.001) for HLA-G*01:04, G*01:03, G*01:05N as compared to VBMD, while HLA-G*01:01 frequency was significantly higher in VBMD than in Malians (p<0.001); no statistical difference was found for G*01:06 (Table 1).

Frequencies of SNPs in the 5′URR and 3′UTR were significantly different between VBMD and Malians (p<0.05), except for −725 C.

#### Hardy Weinberg Equilibrium and Linkage Disequilibrium

All SNPs of the HLA-G gene were in Hardy Weinberg Equilibrium (HWE), except the SNP at codon 130 that displayed a significantly higher heterozygosity than expected. A T in codon 130 specifically defines the HLA-G*01:05N allele.

**Figure 1:**
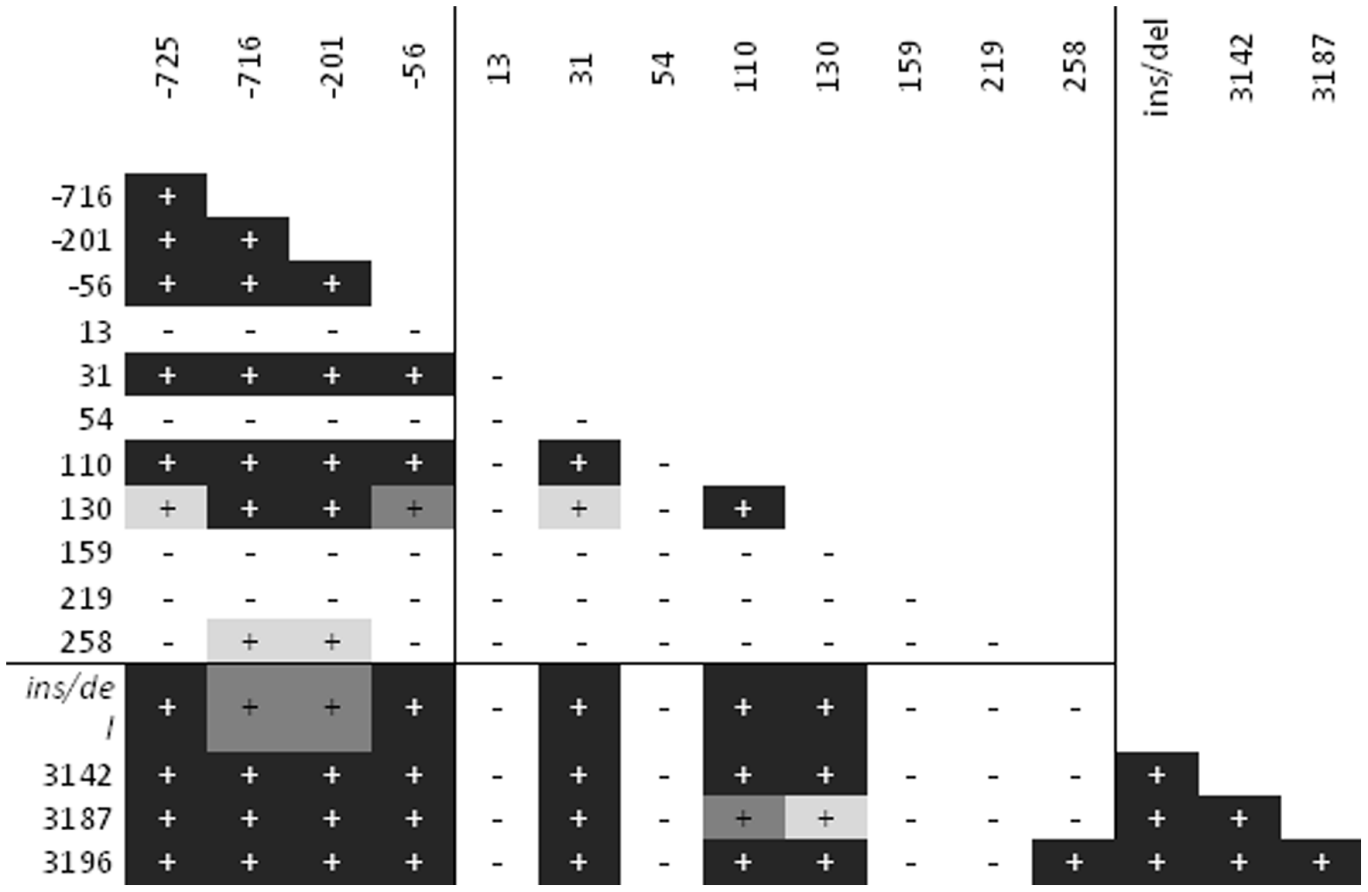
Two-locus linkage disequilibrium (LD) in the Malian population (n = 230) between polymorphisms in the 5′URR (−725 C/G or T; −716 G/T; −201 G/A and −56 C/T), in the coding region (codons 13C/T; 31 A/T; 54 A/G; 110 C/A; 130 C/T; 159 T/C; 219 C/T and 258 C/T) and in the 3′UTR (*ins/del* exon 8; 3142 C/G; 3187 G/A and 3196 C/G). Statistical significance (p value at junction between two loci) is indicated by color (light gray: p<0.05, gray: p<0.01 and black p<0.001).

Two-locus Linkage Disequilibrium (LD) for SNPs in 5′URR, 3′UTR and coding alleles is shown in Figure 1. Strong LD was observed between SNPs within each non-coding region and between the SNPs of both 5′URR and 3′UTR. A strong LD was also present between all 5′URR and 3′UTR SNPs and codon 31 (defining allele HLA-G*01:03), codon 110 (defining HLA-G*01:04) and codon 130 (defining HLA-G*01: 05N). Codon 258 (defining HLA-G*01:06) was in LD with −725 and −201 positions. LD was observed between codon 31, codon 110 and codon 130. Malians displayed higher levels of LD compared to VBMD among and between the SNPs in 5′URR, 3′UTR and the coding regions.

Global LD was observed between HLA-A and HLA-G alleles and between HLA-A alleles and HLA-G UTR, respectively (both p = 0 and quantile = 1000).

#### UTR and HLA-G∼UTR haplotypes

UTR composition, their allelic association and their frequencies in the Malian samples are shown in Table 2. These results are compared with those obtained in French VBMD [41].

**Table 2:** Description of the UTR haplotypes in the Malian population: nomenclature, polymorphism composition, estimated frequencies (Fq) with EM algorithm and association with coding HLA-G alleles.

UTR haplotype	−725	−716	−201	−56	ex 8	3142	3187	3196	Coding allele	Ratio	Fq Malians	*Fq VBMD * [41]	*P value*
**UTR-1**	C	T	G	C	*del*	C	G	C	G*01:01	1	0.134	*0.336*	***
**UTR-2**	**C**	G	A	C	*ins*	G	A	G	G*01:01	0.563	0.165	*0.152*	NS
									G*01:05N	0.375	0.109	*0.021*	***
									G*01:06	0.083	0.025	*0.033*	NS
**UTR-3**	C	G	A	C	*del*	G	A	C	G*01:04	1	0.239	*0.123*	***
**UTR-4**	G	T	G	C	*del*	C	A	C	G*01:01	1	0.05	*0.156*	***
**UTR-5**	T	T	G	T	*ins*	G	A	C	G*01:03	1	0.143	*0.028*	***
**UTR-6**	C	T	G	C	*del*	C	A	C	G*01:01	1	0.121	*0.074*	NS

Comparison with previously published results on VBMD [41]. Statistical differences between the two population frequencies were measured using chi-squared test (NS: no significant; *: p<0.05; **: p<0.01; ***: p<0.001).

UTR haplotypes, defined by four SNPs in the 5′URR (−725 C/G/T, −716 G/T, −201 G/A and −56 C/T) and four SNPs in the 3′UTR (*ins/del* exon 8, 3142 C/G, 3187 G/A and 3196 C/G), are restricted to a small number in the Malian samples (6 UTRs estimated out of 28 possible) (Table 2). In comparison with VBMD, the same UTRs were estimated, but with a lower diversity in the Malian samples, as UTR-7 and UTR-8 previously reported for VBMD were not detected in the Malian samples. No significant difference was observed for UTR frequencies, either between villages or between ethnic groups.

UTR-2 had the highest frequency in the Malian samples. UTR frequencies were as follows: UTR-2 (29.9%)>UTR-3 (23.9%)>UTR-5 (14.3%)>UTR-1 (13.4%)>UTR-6 (12.1%)>UTR-4 (5%). Malians displayed significantly higher frequencies (p<0.001) for UTR-2, UTR-3 and UTR-5 compared to VBMD and lower frequencies for UTR-1 and UTR-4 (p<0.001).

Concerning the association between UTR and HLA-G alleles, the same association previously reported in the VBMD population was described in the Malian samples (Table 2). UTR-3∼G*01:04 showed the highest frequencies in Malians followed by UTR-2∼G*01:01, UTR-5∼G*01:03, UTR-1∼G*01:01, UTR-6∼G*01:01, UTR-2∼G*01:05N, UTR-4∼G*01:01 and UTR-2∼G*01:06. Significant differences between Malians and the French are shown in Table 2; notably, UTR-5∼G*01:03 and UTR-2∼G*01:05N display significant different frequencies.

#### HLA-A∼UTR∼HLA-G haplotypes

Haplotype analysis was extended to HLA-A alleles in association with UTR and HLA-G alleles. These HLA-A∼UTR∼HLA-G haplotypes are in a limited number (43 HLA-A∼UTR∼HLA-G estimated out of 204 possible). HLA-A∼UTR∼HLA-G haplotypes estimated in the Malian samples and their frequencies are showed in Table S1. Notably, the two HLA-A∼UTR∼HLA-G haplotypes displaying the highest frequency are G01:04∼UTR3∼A23:01:01 (16.5%) and G01:05N∼UTR2∼A30:01:01 (10.3%). Interestingly, haplotypes A23:01:01∼G01:04 (10.7), A30:01:01∼G01:05N (15.5), A23:01:01∼UTR3 (11.1) and A30:01:01∼UTR2 (7.7) display high standardized residuals (Table S2).

Association between HLA-A and HLA-G was further investigated using the Median Joining (MJ) method based on protein sequences (Figure S1). UTR were listed for informative purposes as the network is based on protein sequences. The resulting network revealed an HLA-A clustering in accordance with other studies [45], [46], [47]. The five HLA-A lineages previously defined by Gu et al. [45] are also indicated. Most of the HLA-G alleles and UTR were distributed throughout HLA-A lineages however HLA-G*01:06∼UTR-2 and HLA-G*01:05N∼UTR-2 were exclusively associated with HLA-A*01:01 and HLA-A*30:01, respectively; but as HLA-G*01:06 and HLA-G*01:05N alleles are the least frequent, other associations may not have been detected.

### Serology

The overall distribution of sHLA-G fits a Gaussian distribution. The sHLA-G mean value in the 219 plasma samples was 143.18±31.05 UI/ml.

#### Influence of age and gender on sHLA-G

No significant difference was found between gender and sHLA-G level even though men displayed lower values than women (139.7±29.87 UI/ml vs 147.4±32.06 UI/ml, p = 0.169).

**Figure 2:**
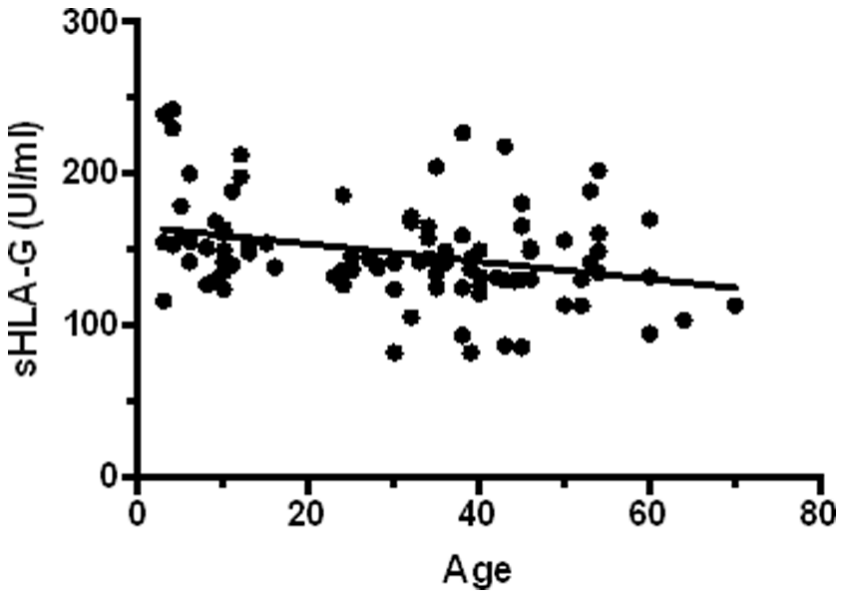
Correlation between sHLA-G levels and age in women. Statistical correlation between age and sHLA-G levels was tested using Spearman's rank test (rS = −0.1905, p = 0.004).

**Figure 3:**
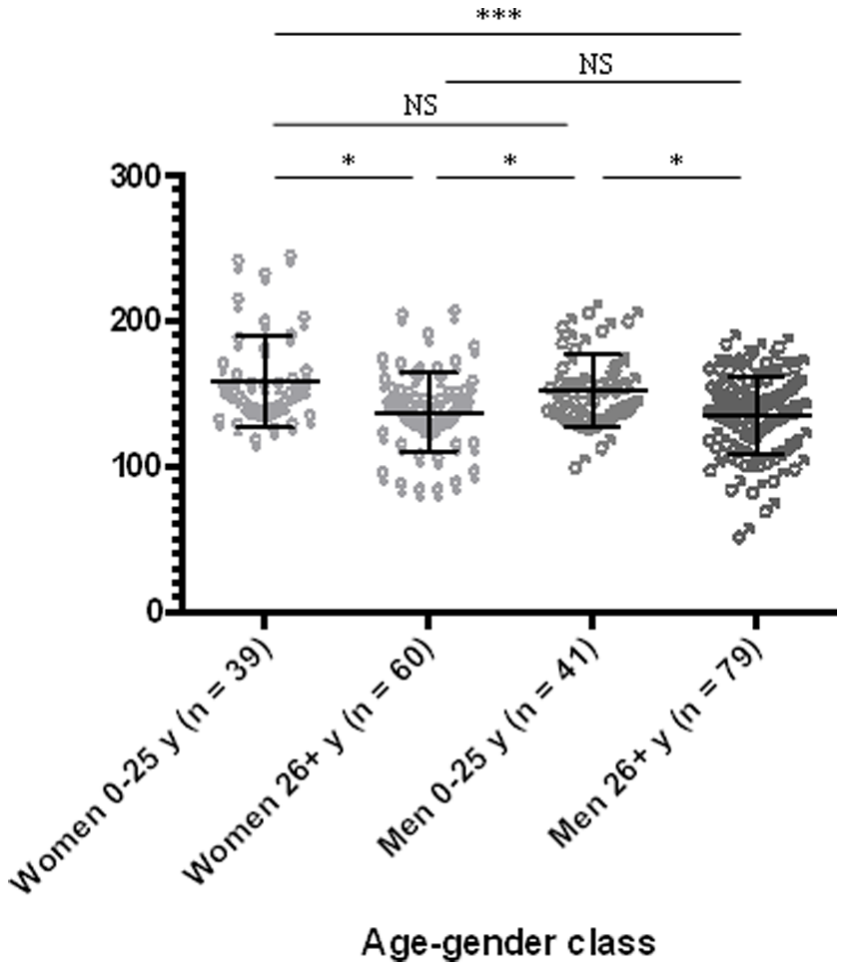
Comparison between two groups of women (3–25 years old n = 39 and over 26 years old n = 60) and men (3–25 years old n = 41 and over 26 years old n = 79) related to sHLA-G levels. Statistical comparison was based on Kruskal-Wallis one-way ANOVA followed by Dunn post-hoc test (girls 3–25 years old: 158.4±31.6 UI/ml; boys 3–25 years old: 152.0±31.6 UI/ml; women over 26 years old: 137.5±26.6 UI/ml; men over 26 years old: 135.5±26.4 UI/ml). NS: not significant; *: p>0.05; **: p<0.01; ***: p<0.001).

However, a significant negative correlation (rS = −0.206, p = 0.002) was observed between age and sHLA-G levels. When individuals were classified according to gender, a significant negative correlation was only found for women (rS = −0.1905, p = 0.004) (Figure 2). Both boys and girls between 3–25 years old showed statistically higher sHLA-G values than men and women over 26 years old (p<0.05; Figure 3).

#### Influence of 5′ URR and 3′UTR SNPs and HLA-G alleles on sHLA-G

Significant associations were found between 5′URR −716 G/T (p = 0.03), −201 G/A (p = 0.03) and 3′UTR +3196 C/G (p = 0.03) and sHLA-G. The Dunn post-hoc test showed significantly higher sHLA-G levels for −716 T/T; −201 G/G and +3196 C/C genotypes. No significant associations were observed for HLA-G alleles and the other 5′URR and 3′UTR SNPs.

The exon 8 *ins/ins* genotype did not show significantly lower sHLA-G than ins/del or del/del genotypes, thus confirming our previous results [41] (*ins/ins* 136±30.67 UI/ml, all except *ins/ins* 145±31.0 UI/ml, p = 0.144).

#### Association between HLA-G UTR haplotypes and sHLA-G

sHLA-G mean values and standard deviation for each UTR genotype are shown in Table S3.

**Figure 4:**
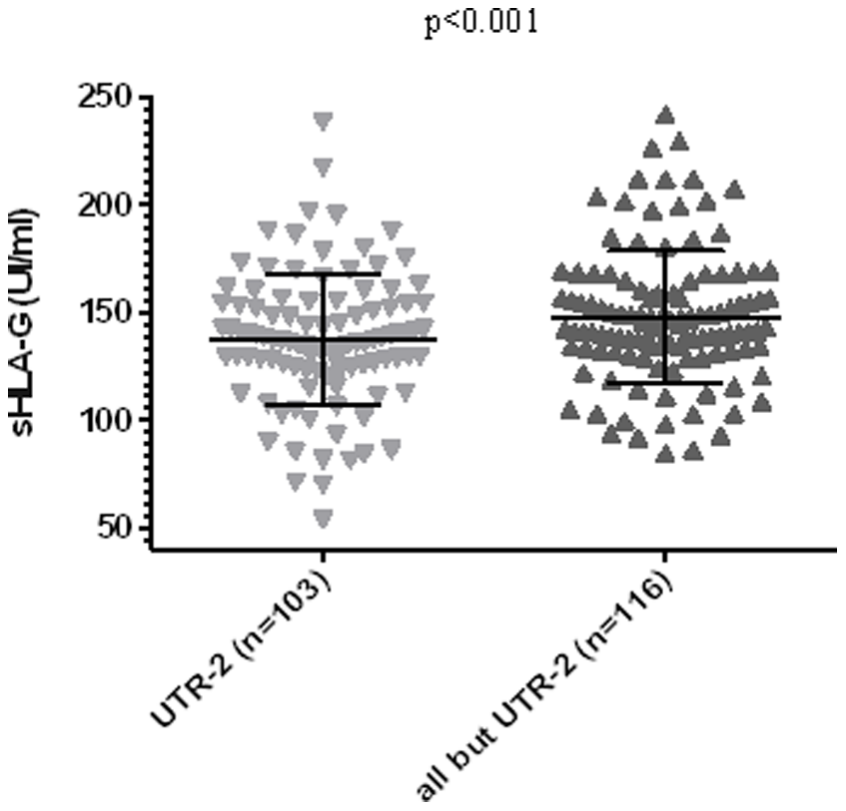
Comparison between UTR-2 (n = 103) and non-UTR-2 (n = 116) individuals related to sHLA-G production. Statistical comparison was based on Mann-Whitney t-test. (UTR-2 137.5±30.6 UI/ml vs. all except UTR-2 148.2±30.7 UI/ml). When six outer points were excluded (238.6 UI/ml, 217.6 UI/ml and 53.8 UI/ml for UTR-2; 242.2 UI/ml, 229.8 UI/ml and 226.5 UI/ml for non-UTR-2) UTR-2 individuals still displayed significantly lower values (p<0.05).

**Figure 5:**
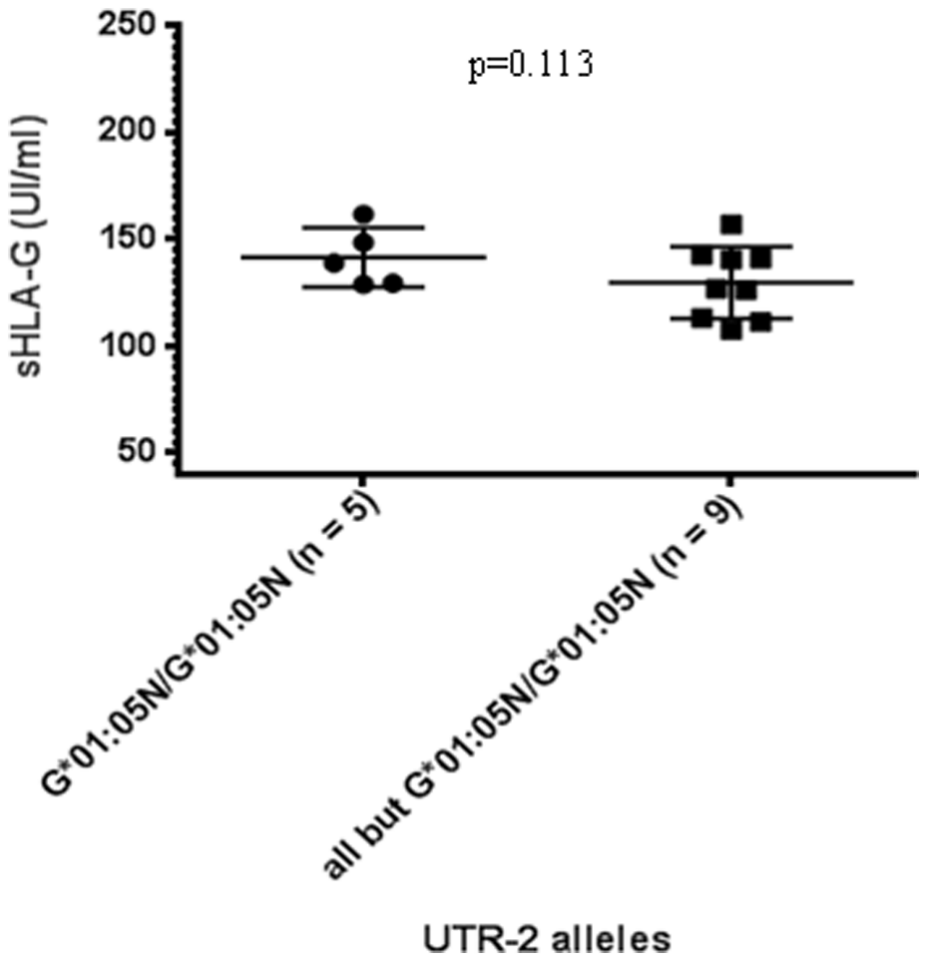
Comparison between UTR-2 alleles G*01:05N/G*01:05N (n = 5 141.4±13.9 UI/ml) and all except G*01:05N/G*01:05N (n = 9 129.5±16.8 UI/ml). Statistical comparison was based on Kruskal-Wallis one-way ANOVA followed by Dunn post-hoc test.

UTR-2 individuals displayed a significantly lower level of sHLA compared to non-UTR-2 individuals (Figure 4; 137.5±30.6 UI/ml vs. 148.2±30.7 UI/ml, p<0.01). This difference remained significant even when the outer points were not taken into account. As UTR-2 is associated with three different HLA-G alleles (i.e. HLA-G*01:01, G*01:05N and G*01:06), an allelic effect could mask the low sHLA-G status of UTR-2 individuals. HLA-G*01:05N/G*01:05N individuals showed higher sHLA-G levels compared to the other UTR-2 alleles (Figure 5), but this difference was not significant (141.4±13.9 UI/ml vs. 129.5±16.8 UI/ml, p = 0.113)

No significant correlation was found between sHLA-G and the other UTRs.

#### Association between HLA-A and sHLA-G

**Figure 6:**
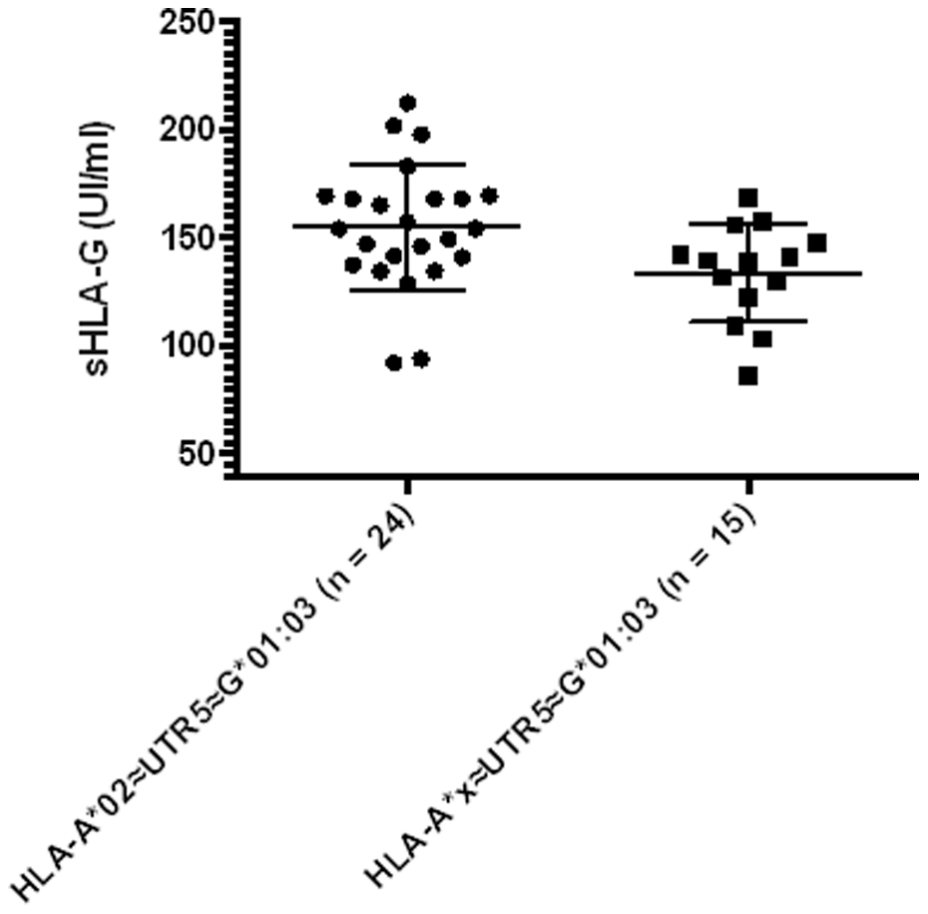
Comparison between A*02≈UTR-5≈G*01:03 (n = 24) and A*x≈UTR-5≈G*01:03 (n = 15, x corresponds to all HLA-A alleles except A*02). Statistical comparison was based on Mann-Whitney t-test (A*02≈UTR-5≈G*01:03 154.9±28.9 UI/ml; A*x≈UTR-5≈G*01:03 133.9±22.5 UI/ml). When outer points were excluded (212.4 UI/ml, 201.9 UI/ml, 92.2 UI/ml and 92.2 UI/ml for A*02≈UTR-5≈G*01:03; 86.4 UI/ml for A*x≈UTR-5≈G*01:03), the difference remained significant (p<0.05).

To assess the influence of HLA-A alleles on sHLA-G expression, we tested the correlation within and among the A lineages defined by Gu X et al. [45], taking into consideration or not the association with HLA-G∼UTR haplotypes. Among these, the A-V lineage homozygous HLA-A*02 subtype (associated with UTR-1/UTR-1, UTR-1/UTR-5 or UTR-5/UTR-5; 183.5±25.83 UI/ml) showed significantly higher sHLA-G levels than the HLA-A*68 subtype (associated with UTR-2/UTR-2 and UTR-2/UTR-5; 117.2±33.51 UI/ml) (Figure S2, p = 0.002). Following a previously reported hypothesis that UTR-5 may be split into two distinct subgroups respectively associated with high and low sHLA-G expression [41], we wanted to assess the possible influence of HLA-A specific subtypes. The HLA-A*02 subtypes bearing UTR-5 displayed significantly higher sHLA-G levels compared to the other HLA-A subtypes associated with UTR-5 (HLA-A*02 UTR-5 154.9±28.88 UI/ml; HLA-A*X UTR-5 133.9±22.55 UI/ml; p = 0.024; Figure 6).

No significant differences in sHLA-G expression were found between HLA-A alleles from other HLA-A lineages.

## Discussion

### HLA-G haplotypes conservation between different populations

In this study we present results based on HLA-G and HLA-A genotypes and sHLA-G serological analyses performed on 229 Malian samples. UTR haplotypes described in the Malian samples show reduced diversity (n = 6 estimated with the EM algorithm among 28 possible) and share a similar structure to those described in the French VBMD and in the Brazilian population [24], [41]. The Malian samples show less diversity than the southeastern French samples for the chosen genetic polymorphisms. These results suggest that UTR haplotype structure is conserved between geographically distant populations despite distinct demographic histories and environments. Although UTR structure is conserved, there are significant differences in UTR frequency between the French and the Malians. The Malians have a significantly higher frequency of UTR-2∼G*0105N, UTR-3∼G*01:04 and UTR-5∼G*01:03 than the VBMD. Interestingly, UTR-5 revealed a large difference between the two populations. We expected to find these results as UTR-5 is associated with HLA-G*01:03 and our team previously showed that the frequency of this allele greatly varies according to location, i.e. from 1.6% in southeast France to 9% in Congo-Brazzaville [48]. Based on these findings, we had speculated that UTR-5 frequency would be higher in sub-Saharan populations. UTR-2 and UTR-3 are associated respectively with HLA-G*01:05N and HLA-G*01:04. These alleles reach frequencies of up to 10% in a population of Zimbabwe Shona [49].

Stronger LD between SNPs within 5′URR and 3′UTR was found in the Malian samples than in the French samples. Previous multi-loci studies have suggested that sub-Saharan Africans display a lower LD compared to populations from other continents [50], [51], [52]. Furthermore, LD between codon 31, codon 110 and codon 130 was only observed in Malians. This LD can be explained by the fact that codon 31 (T), codon 110 (A) and codon 130 (A) are specific to HLA-G*01:03, G*01:04 and G*01:05N, respectively, which, as previously mentioned, display frequencies of over 10% in the Malian population.

Haplotype structure conservation was further confirmed when HLA-A alleles were considered since the number of HLA-A∼UTR∼HLA-G haplotypes was also greatly reduced in the Malian samples. The estimated haplotypes were limited to 43 compared to the 204 possible haplotypes according to the observed alleles. One should keep in mind that we studied 229 samples and that some rare haplotypes might not have been detected.

### Impact of genetics on sHLA-G expression

The second objective of this study was to confirm the correlation between sHLA-G expression level and UTR haplotypes. Highly variable sHLA-G values have been reported with the Elisa kit using the MEM-G/9 antibody according to the biological fluid analyzed or the calibration standard used; our results are in accordance with published data based on plasma samples and expressing their results in U/ml according to the calibration standard displayed by the supplier [53], [54].

We confirmed an association between the UTR-2 haplotype and lower sHLA-G levels in the Malian samples. UTR-2 individuals showed, both in homozygous and heterozygous state, a significant association with lower values. This result tends to show a Dominant Negative Effect (DNE) of UTR-2. Moreover, even though no significant association was observed between HLA-G alleles and sHLA-G levels, an allelic effect was still observed for UTR-2. Indeed, HLA-G*01:05N/G*01:05N showed higher (but not significantly) sHLA-G levels compared to other UTR-2 allelic combinations. This putative allelic effect of HLA-G*01:05N/G*01:05N on sHLA-G does not exclude a haplotype effect of HLA-A on UTR-2; indeed HLA-G*01:05N∼UTR-2 was only coupled with HLA-A*30:01. However, HLA-G*01:05N codes for a truncated protein since HLA-G*01:05N presents a stop codon in position +189, therefore the mRNA of G*01:05N translates only HLA-G5 and -G6 soluble isoforms [27]. Moreover, the ELISA technique used in this study uses a monoclonal antibody MEM-G/9 that only detects the HLA-G1 and HLA-G5 isoforms. Thus, the outlines for an explanation could be that the HLA-G*01:05N/G*01:05N genotype displays a dose effect that may interfere with the impact of UTR-2 on sHLA-G expression. As recently suggested, an equilibrium between membrane and soluble HLA-G has to be reached by the cell but in the case of HLA-G*01:05N there may be an over production of sHLA-G, since this isoform cannot bind to the membrane [55]. Thus, to assess the clinical relevance of UTR-2, the associated HLA-G alleles should be taken into account. For instance, in Recurrent Spontaneous Abortions (RSA), for which results on correlation with HLA-G*01:05N are contradictory [28], [56], [57], [58], verification of the HLA-G*01:05N/G*01:05N effect highlighted in the present study might be helpful.

No significant correlation has been found between the other UTRs and sHLA-G levels. However, UTR-1 and UTR-5 homozygous individuals displayed higher sHLA-G levels than the overall sample mean. Interestingly HLA-A*02 subtypes bearing UTR-5 displayed significantly higher sHLA-G levels compared to other HLA-A alleles bearing UTR-5. This differential sHLA-G expression may be due to an HLA-A*02 effect or to supplementary polymorphisms in regulatory regions of UTR-5. Indeed, 3 SNPs in the 5′URR, −646, −540 and −509 described by Castelli et al. (2011) can separate UTR-5 in 2 sub haplotypes [24]. One of these sub haplotypes could be associated to higher sHLA-G levels.

Our analyses on HLA-A showed that HLA-A alleles display LD with both HLA-G alleles and UTR. HLA-A*23:01:01∼HLA-G*01:04∼UTR3 displayed the highest frequency, confirming the reported LD between HLA-A*23 (and HLA-A*24) and HLA-G*01:04 in different populations [36], [59]. HLA-A*23:01 reaches high frequencies in sub-Saharan Africans, notably in the Malian Bandiagara population [60]. HLA-G*01:04 has been reported with moderate to high frequencies in several sub-Saharan African populations, i.e. Zambians, Ghanaians, Pygmies and Congolese [48], [49], [61]. HLA-A*23 and HLA-A*24 are associated with a large-scale deletion of 50 kb including the region that precedes HLA-G [34], [38], [39], [40]. This strong LD observed could be emphasized by a deletion occurring between those two loci, thus reducing the recombination rate between HLA-A and HLA-G.

HLA-G*01:04 has been previously associated with a high sHLA-G production [26]. Here, neither HLA-G*01:04 nor HLA-A*23:01∼HLA-G*01:04∼UTR3 showed any significant difference in sHLA-G levels. Several explanations are possible, for example the high levels of sHLA class-I molecules reported in the serum of HLA-A*23 and HLA-A*24 individuals might have cross-reacted with the HLA-G antibody [62], [63], [64], [65]. Interestingly it has been suggested that the HLA-A*23:01∼HLA-G*01:04 haplotype may constitute a risk factor for allograft rejection in renal transplantation [66]. Interaction with other immune effectors could also be incriminated; for instance HLA-E, another immunosuppressive molecule which can modulate various immune competent cells such as NK cells and T lymphocytes. We showed in a previous study that there is not significant LD between HLA-G and HLA-E in Southeastern French and Teke Congolese, while two haplotypes in Tswa Pygmies, i.e. HLA-G*01:04∼E*01:03:01 and G*01:04∼E*01:01, exhibited highly significant positive and negative LD values respectively [48].

### sHLA-G level is potentially age and gender dependent

This study showed a negative correlation between age and sHLA-G levels in Malian women which remained significant when UTR-2 individuals were excluded. None of the previously published studies have shown correlation between age and sHLA-G levels, even related with gender. Moreover, we also found that boys and girls between 3–25 years old showed statistically higher sHLA-G values compared to men and women over 26 years old. It has recently been reported that mesenchymal progenitors and osteoblastic cells specifically express HLA-G5 during osteogenesis, with a key role in bone homeostasis [67]. Therefore the different pattern of sHLA-G expression observed between growing individuals and mature individuals might be linked to osteoblast expression, involved in the development, growth and remodelling of bones. The conservation of the dominant negative effect of UTR-2 on sHLA-G expression among different age groups suggests that the regulation mechanism of sHLA-G expression might be independent to UTR. Furthermore the negative correlation between age and sHLA-G specific to women may also be linked to progesterone secretion or other sexual hormones; it was reported that expression of sHLA-G can be induced by progesterone [68], [69], [70]. As this hormone in women drops to levels lower than in men after menopause, it may induce a wider gap between younger and older women, highlighted by a negative correlation between age and sHLA-G.

## Conclusion

Taken together, these data support the theory of a conservation of UTR haplotype structure in populations with different origins and demographic history, such as Malians, French and Brazilians. These UTR haplotypes appear to be implicated in different sHLA-G expression patterns. Particularly, the association between the UTR-2 haplotype and low sHLA-G levels seems to be further confirmed and preserved in different populations, displaying a dominant negative effect of UTR-2. However, the allelic effect of HLA-G and HLA-A genes, independent from UTRs, seems to be implicated in sHLA-G modulation. Moreover, results on age and gender indicate that both of these parameters should be further investigated in studies involving sHLA-G expression. Finally these data suggest that sHLA-G production is not only regulated by UTR but also potentially by specific microenvironments. For exemple, UTR-1 and UTR-3 have been associated with different levels of malaria infection under a recessive model [71], suggesting that HLA-G expression may have a predictive value for parasite infection outcome. Thus, one of the limitations of this study is not to have taken into account the influence of malaria infection on sHLA-G expression.

These results may constitute essential elements on the one hand to optimize the selection of donors for organ transplantation and on the other hand the diagnosis and treatment of infectious and parasitic diseases. Further investigations in larger cohorts and in different populations are necessary not only to detect new functional polymorphisms in HLA-G regulatory regions, but also to reveal the extent of biological phenomena that influence sHLA-G secretion.

## Supporting Information

Figure S1 Median Joining (MJ) network on HLA-A∼HLA-G haplotypes constructed using the Network program(www.fluxus-engineering.com/network) based on protein sequences (taking into account the first two allele digits). UTR were added afterwards based on estimates for HLA-A∼HLA-G∼UTR haplotypes. Branch length represents the phylogenetic distance between HLA-A∼HLA-G haplotypes based on amino acid substitutions. The yellow circles represent haplotype frequencies. HLA-A lineages based on Gu X. and Nei M. 1999 [45] are indicated. Lineage A-I corresponds to A23 and A24 subtypes; lineage A-II corresponds to A01, A03, A30, A36, and A80 subtypes; lineage A-III corresponds to A29, A32, A33, and A74 subtypes; lineage A-IV corresponds to A26 and A34 subtypes; and lineage A-V corresponds to A02, A68, and A69 subtypes.
(TIF)

Figure S2 Comparison between homozygous HLA-A*02 (associated to UTR-1/UTR-1, UTR-1/UTR-5 or UTR-5/UTR-5; mean = 183.5±25.83 UI/ml) and HLA-A*68 (associated to UTR-2/UTR-2 and UTR-2/UTR-5; mean 117.2±33.51 UI/ml) (p = 0.002).
(TIF)

Table S1 HLA-A∼UTR∼HLA-G haplotype frequencies (Fq) estimated with the Gene[Rate] program in the Malian samples.
(DOCX)

Table S2 Observed (Obs) and expected (Exp) frequencies, differences (Diff) and standardized residuals (StdRes) for HLA-A and HLA-G and for HLA-A and UTR.
(DOCX)

Table S3 Soluble HLA-G mean and standard deviation (SD) are shown for each HLA-G UTR genotype.
(DOCX)
