# Supplementary figures and images for: HLA-G UTR Haplotype Conservation in the Malian Population: Association with Soluble HLA-G
Source: PLoS One. 2013 Dec 23;8(12):e82517. doi: 10.1371/journal.pone.0082517 (PMC3871591; doi:10.1371/journal.pone.0082517)

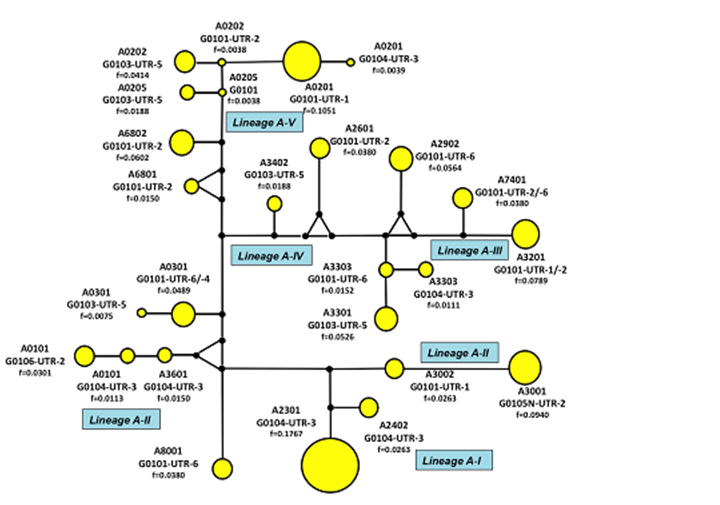

Supplement: Figure S1 — Median Joining (MJ) network on HLA-A∼HLA-G haplotypes constructed using the Network program(www.fluxus-engineering.com/network) based on protein sequences (taking into account the first two allele digits). UTR were added afterwards based on estimates for HLA-A∼HLA-G∼UTR haplotypes. Branch length represents the phylogenetic distance between HLA-A∼HLA-G haplotypes based on amino acid substitutions. The yellow circles represent haplotype frequencies. HLA-A lineages based on Gu X. and Nei M. 1999 [45] are indicated. Lineage A-I corresponds to A23 and A24 subtypes; lineage A-II corresponds to A01, A03, A30, A36, and A80 subtypes; lineage A-III corresponds to A29, A32, A33, and A74 subtypes; lineage A-IV corresponds to A26 and A34 subtypes; and lineage A-V corresponds to A02, A68, and A69 subtypes. (TIF) [file pone.0082517.s001.tif]

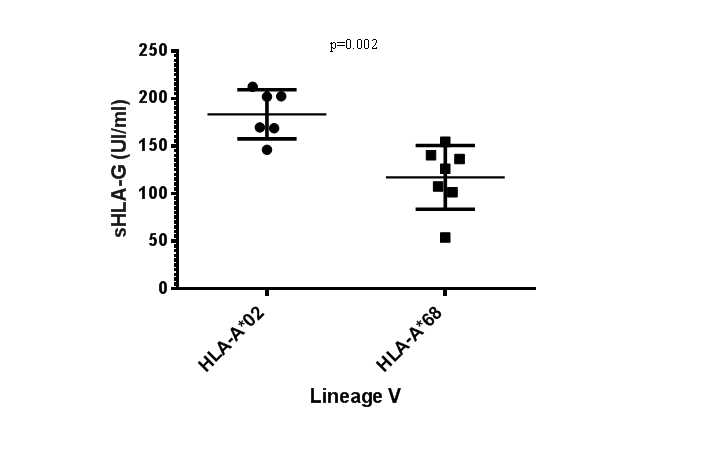

Supplement: Figure S2 — Comparison between homozygous HLA-A*02 (associated to UTR-1/UTR-1, UTR-1/UTR-5 or UTR-5/UTR-5; mean = 183.5±25.83 UI/ml) and HLA-A*68 (associated to UTR-2/UTR-2 and UTR-2/UTR-5; mean 117.2±33.51 UI/ml) (p = 0.002). (TIF) [file pone.0082517.s002.tif]
