# Supplementary material for: HLA-G UTR Haplotype Conservation in the Malian Population: Association with Soluble HLA-G
Source: PLoS One. 2013 Dec 23;8(12):e82517. doi: 10.1371/journal.pone.0082517 (PMC3871591; doi:10.1371/journal.pone.0082517)
Supplement: Table S1 — HLA-A∼UTR∼HLA-G haplotype frequencies (Fq) estimated with the Gene[Rate] program in the Malian samples. (DOCX) [file pone.0082517.s003.docx]

**Table S1.** HLA-A~UTR~HLA-G haplotypes frequencies (Fq) estimated with the Gene[Rate] program in the Malian samples.

| **Haplotype** | **Fq** |
| --- | --- |
| **G01:04~UTR3~A23:01:01** | 0.1651 |
| **G01:05N~UTR2~A30:01:01** | 0.1031 |
| **G01:01~UTR1~A02:01:01:01** | 0.0987 |
| **G01:01~UTR2~A32:01:01** | 0.0679 |
| **G01:01~UTR2~A68:02:01:01** | 0.057 |
| **G01:03~UTR5~A02:02** | 0.0552 |
| **G01:01~UTR6~A29:02:01:01** | 0.0537 |
| **G01:03~UTR5~A33:01:01** | 0.0535 |
| **G01:01~UTR4~A03:01:01:01** | 0.0456 |
| **G01:01~UTR6~A80:01** | 0.0283 |
| **G0106~UTR2~A01:01:01:01** | 0.0252 |
| **G01:04~UTR3~A24:02:01:01** | 0.0252 |
| **G01:01~UTR1~A30:02:01** | 0.0231 |
| **blank~blank~blank** | 0.0224 |
| **G01:01~UTR6~A74:01** | 0.022 |
| **G01:04~UTR3~A01:01:01:01** | 0.0189 |
| **G01:03~UTR5~A02:05:01** | 0.0157 |
| **G01:03~UTR5~A34:02:01** | 0.0157 |
| **G01:04~UTR3~A36:01** | 0.0157 |
| **G01:01~UTR2~A68:01:01:01** | 0.0126 |
| **G01:04~UTR3~A33:03:01** | 0.0094 |
| **G01:01~UTR6~A33:03:01** | 0.0094 |
| **G01:01~UTR2~A74:01** | 0.0063 |
| **G01:01~UTR2~A26:01:01** | 0.0063 |
| **G01:01~UTR1~A32:01:01** | 0.0063 |
| **G01:03~UTR5~A03:01:01:01** | 0.0063 |
| **G01:04~UTR3~A02:01:01:01** | 0.0032 |
| **G01:01~UTR2~A01:02** | 0.0031 |
| **G01:01~UTR2~A02:02** | 0.0031 |
| **G01:01~UTR1~A23:01:01** | 0.0031 |
| **G01:03~UTR5~A33:05** | 0.0031 |
| **G01:01~UTR5~A02:05:01** | 0.0031 |
| **G01:01~UTR3~A33:03:01** | 0.0029 |
| **G01:01~UTR2~A66:01** | 0.0023 |
| **G01:01~UTR6~A03:01:01:01** | 0.0023 |
| **G01:01~UTR4~A74:01** | 0.0016 |
| **G01:01~blank~A66:01** | 0.0009 |
| **blank~UTR6~A03:01:01:01** | 0.0008 |
| **G01:01~blank~A74:01** | 0.0007 |
| **blank~UTR4~A74:01** | 0.0006 |
| **blank~UTR3~A33:03:01** | 0.0003 |
| **blank~blank~A74:01** | 0.0002 |
| **blank~UTR1~A23:01:01** | 0.0001 |
