# Supplementary material for: HLA-G UTR Haplotype Conservation in the Malian Population: Association with Soluble HLA-G
Source: PLoS One. 2013 Dec 23;8(12):e82517. doi: 10.1371/journal.pone.0082517 (PMC3871591; doi:10.1371/journal.pone.0082517)
Supplement: Table S2 — Observed (Obs) and expected (Exp) frequencies, differences (Diff) and standardized residuals (StdRes) for HLA-A and HLA-G and for HLA-A and UTR. (DOCX) [file pone.0082517.s004.docx]

**Table S2.** Observed (Obs) and expected (Exp) frequencies, differences (Diff) and standardized residuals (StdRes) for HLA-A and HLA-G and for HLA-A and UTR.

| **Haplotype** | **Obs** | **Exp** | **Diff** | **StdRes** |
| --- | --- | --- | --- | --- |
| **A23:01:01~G01:04** | 0.1633 | 0.0403339 | 0.12296614 | 10.69607139 |
| **A30:01:01~G01:05N** | 0.1023 | 0.0108931 | 0.09140689 | 15.53238554 |
| **A02:01:01:01~G01:01** | 0.0981 | 0.0480972 | 0.05000276 | 3.966831488 |
| **A32:01:01~G01:01** | 0.0726 | 0.034423 | 0.038177 | 3.605657374 |
| **A68:02:01:01~G01:01** | 0.0565 | 0.0267312 | 0.02976876 | 3.203178523 |
| **A02:02~G01:03** | 0.055 | 0.0087673 | 0.04623271 | 8.766334485 |
| **A33:01:01~G01:03** | 0.0535 | 0.0080732 | 0.04542685 | 8.979341628 |
| **A29:02:01:01~G01:01** | 0.0533 | 0.0252119 | 0.02808812 | 3.114502831 |
| **A03:01:01:01~G01:01** | 0.0486 | 0.0252119 | 0.02338812 | 2.593351423 |
| **blank~blank** | 0.0298 | 0 | 0.0298 |  |
| **A74:01~G01:01** | 0.0293 | 0.0139116 | 0.01538836 | 2.310333357 |
| **A80:01~G01:01** | 0.0283 | 0.0134368 | 0.01486316 | 2.271112183 |
| **A01:01:01:01~G01:06** | 0.0252 | 0.0011088 | 0.0240912 | 12.89449915 |
| **A24:02:01:01~G01:04** | 0.0252 | 0.0061009 | 0.01909908 | 4.347098561 |
| **A30:02:01~G01:01** | 0.0229 | 0.0108254 | 0.01207456 | 2.058252087 |
| **A01:01:01:01~G01:04** | 0.0189 | 0.0106524 | 0.0082476 | 1.417398175 |
| **A02:05:01~G01:03** | 0.0157 | 0.002852 | 0.01284799 | 4.284035263 |
| **A34:02:01~G01:03** | 0.0157 | 0.0023691 | 0.01333087 | 4.878233079 |
| **A36:01~G01:04** | 0.0157 | 0.003801 | 0.01189903 | 3.435191526 |
| **A33:03:01~G01:01** | 0.0127 | 0.0104456 | 0.0022544 | 0.391289034 |
| **A68:01:01:01~G01:01** | 0.0126 | 0.0059825 | 0.00661752 | 1.521126087 |
| **A33:03:01~G01:04** | 0.0093 | 0.0053262 | 0.0039738 | 0.968391948 |
| **A26:01:01~G01:01** | 0.0063 | 0.0029912 | 0.00330876 | 1.077215724 |
| **A03:01:01:01~G01:03** | 0.0063 | 0.0080128 | -0.0017128 | -0.339843396 |
| **A23:01:01~blank** | 0.004 | 0 | 0.004 |  |
| **A02:01:01:01~G01:04** | 0.0033 | 0.0245247 | -0.0212247 | -2.387051279 |
| **A02:02~G01:01** | 0.0031 | 0.0275859 | -0.0244859 | -2.592456534 |
| **A66:01~G01:01** | 0.0031 | 0.0014719 | 0.00162812 | 0.756212617 |
| **A01:02~G01:01** | 0.0031 | 0.0014719 | 0.00162812 | 0.756212617 |
| **A33:05~G01:03** | 0.0031 | 0.0004678 | 0.00263221 | 2.16973665 |
| **A02:05:01~G01:01** | 0.0031 | 0.0089737 | -0.0058737 | -1.100735893 |
| **A29:02:01:01~G01:03** | 0 | 0.0080128 | -0.0080128 | -1.589858513 |
| **A30:01:01~G01:01** | 0 | 0.0483821 | -0.0483821 | -3.826373004 |
| **A66:01~G01:05N** | 0 | 0.0003314 | -0.0003314 | -0.324572176 |
| **A02:01:01:01~G01:05N** | 0 | 0.010829 | -0.010829 | -1.845622303 |
| **A30:01:01~G01:04** | 0 | 0.02467 | -0.02467 | -2.766137984 |
| **A33:03:01~G01:05N** | 0 | 0.0023518 | -0.0023518 | -0.863778649 |
| **blank~G01:04** | 0 | 0.0081104 | -0.0081104 | -1.599429263 |
| **A01:01:01:01~G01:01** | 0 | 0.0208912 | -0.0208912 | -2.550414274 |
| **A68:02:01:01~G01:04** | 0 | 0.0136302 | -0.0136302 | -2.067688103 |
| **A32:01:01~G01:04** | 0 | 0.0175523 | -0.0175523 | -2.341718512 |
| **A03:01:01:01~G01:04** | 0 | 0.0128555 | -0.0128555 | -2.008854944 |
| **A02:02~G01:04** | 0 | 0.014066 | -0.014066 | -2.100017629 |
| **A23:01:01~G01:03** | 0 | 0.0251399 | -0.0251399 | -2.791687598 |
| **A02:05:01~G01:04** | 0 | 0.0045757 | -0.0045757 | -1.203499677 |
| **A01:02~G01:03** | 0 | 0.0004678 | -0.0004678 | -0.385600354 |
| **A33:03:01~G01:06** | 0 | 0.0005544 | -0.0005544 | -0.419763576 |
| **A30:01:01~G01:03** | 0 | 0.0153767 | -0.0153767 | -2.194220782 |
| **A34:02:01~G01:05N** | 0 | 0.0016783 | -0.0016783 | -0.729940546 |
| **blank~G01:01** | 0 | 0.0159058 | -0.0159058 | -2.231051805 |
| **A30:02:01~blank** | 0 | 0 | 0 |  |
| **A23:01:01~G01:05N** | 0 | 0.0178095 | -0.0178095 | -2.358510244 |
| **A74:01~G01:03** | 0 | 0.0044214 | -0.0044214 | -1.183122662 |
| **A74:01~G01:05N** | 0 | 0.0031322 | -0.0031322 | -0.996448857 |
| **A26:01:01~G01:03** | 0 | 0.0009507 | -0.0009507 | -0.549568613 |
| **A33:01:01~G01:01** | 0 | 0.0254018 | -0.0254018 | -2.80581226 |
| **A23:01:01~G01:01** | 0 | 0.0791017 | -0.0791017 | -4.812960019 |
| **A68:02:01:01~blank** | 0 | 0 | 0 |  |
| **A68:02:01:01~G01:03** | 0 | 0.0084957 | -0.0084957 | -1.636664584 |
| **A02:02~G01:05N** | 0 | 0.0062109 | -0.0062109 | -1.400998259 |
| **A02:01:01:01~blank** | 0 | 0 | 0 |  |
| **A74:01~blank** | 0 | 0 | 0 |  |
| **A29:02:01:01~blank** | 0 | 0 | 0 |  |
| **blank~G01:05N** | 0 | 0.0035812 | -0.0035812 | -1.065235873 |
| **A30:01:01~blank** | 0 | 0 | 0 |  |
| **A29:02:01:01~G01:06** | 0 | 0.0013381 | -0.0013381 | -0.651884008 |
| **A80:01~G01:06** | 0 | 0.0007132 | -0.0007132 | -0.476049521 |
| **A32:01:01~G01:03** | 0 | 0.0109403 | -0.0109403 | -1.854976651 |
| **A32:01:01~blank** | 0 | 0 | 0 |  |
| **A29:02:01:01~G01:04** | 0 | 0.0128555 | -0.0128555 | -2.008854944 |
| **A02:05:01~blank** | 0 | 0 | 0 |  |
| **A33:01:01~blank** | 0 | 0 | 0 |  |
| **A24:02:01:01~G01:06** | 0 | 0.000635 | -0.000635 | -0.449237663 |
| **A03:01:01:01~blank** | 0 | 0 | 0 |  |
| **A34:02:01~G01:01** | 0 | 0.0074544 | -0.0074544 | -1.533889181 |
| **A32:01:01~G01:06** | 0 | 0.001827 | -0.001827 | -0.761527766 |
| **A80:01~G01:03** | 0 | 0.0042705 | -0.0042705 | -1.1628457 |
| **A80:01~blank** | 0 | 0 | 0 |  |
| **A68:01:01:01~blank** | 0 | 0 | 0 |  |
| **A24:02:01:01~G01:01** | 0 | 0.011965 | -0.011965 | -1.938899769 |
| **A24:02:01:01~G01:03** | 0 | 0.0038027 | -0.0038027 | -1.097567237 |
| **A33:05~G01:04** | 0 | 0.0007505 | -0.0007505 | -0.488347276 |
| **A36:01~G01:01** | 0 | 0.0074544 | -0.0074544 | -1.533889181 |
| **A02:01:01:01~G01:03** | 0 | 0.0152862 | -0.0152862 | -2.187851905 |
| **A68:02:01:01~G01:05N** | 0 | 0.0060185 | -0.0060185 | -1.379258812 |
| **A68:01:01:01~G01:04** | 0 | 0.0030505 | -0.0030505 | -0.983405914 |
| **A74:01~G01:04** | 0 | 0.0070935 | -0.0070935 | -1.496576548 |
| **A34:02:01~G01:04** | 0 | 0.003801 | -0.003801 | -1.097321373 |
| **A03:01:01:01~G01:05N** | 0 | 0.0056764 | -0.0056764 | -1.339718483 |
| **A30:02:01~G01:04** | 0 | 0.0055199 | -0.0055199 | -1.32122393 |
| **A68:01:01:01~G01:03** | 0 | 0.0019013 | -0.0019013 | -0.776837512 |
| **A33:01:01~G01:04** | 0 | 0.0129524 | -0.0129524 | -2.016308148 |
| **A33:03:01~blank** | 0 | 0 | 0 |  |
| **A80:01~G01:05N** | 0 | 0.0030253 | -0.0030253 | -0.979349499 |
| **A24:02:01:01~blank** | 0 | 0 | 0 |  |
| **A26:01:01~G01:05N** | 0 | 0.0006735 | -0.0006735 | -0.462622122 |
| **blank~G01:03** | 0 | 0.0050552 | -0.0050552 | -1.264678361 |
| **A02:02~blank** | 0 | 0 | 0 |  |
| **A68:01:01:01~G01:05N** | 0 | 0.0013469 | -0.0013469 | -0.654025985 |
| **A33:01:01~G01:05N** | 0 | 0.0057192 | -0.0057192 | -1.344726121 |
| **A30:02:01~G01:05N** | 0 | 0.0024373 | -0.0024373 | -0.87930579 |
| **A29:02:01:01~G01:05N** | 0 | 0.0056764 | -0.0056764 | -1.339718483 |
| **A36:01~G01:03** | 0 | 0.0023691 | -0.0023691 | -0.866947794 |
