# Supplementary material for: HLA-G UTR Haplotype Conservation in the Malian Population: Association with Soluble HLA-G
Source: PLoS One. 2013 Dec 23;8(12):e82517. doi: 10.1371/journal.pone.0082517 (PMC3871591; doi:10.1371/journal.pone.0082517)
Supplement: Table S3 — Soluble HLA-G mean and standard deviation (SD) are shown for each HLA-G UTR genotype. (DOCX) [file pone.0082517.s005.docx]

**Table S3.** Soluble HLA-G mean and standard deviation (SD) are shown for each HLA-G UTR genotype.

| **Haplotypes** | **N** | **sHLA-G levels UI/ml** | **SD** |
| --- | --- | --- | --- |
| **UTR-1/UTR-1** | 3 | 158.8 | 49.4 |
| **UTR-2/UTR-2** | 17 | 137.9 | 27.0 |
| **UTR-3/UTR-3** | 14 | 151.1 | 45.8 |
| **UTR-4/UTR-4** | 2 | 174.3 | 36.0 |
| **UTR-5/UTR-5** | 5 | 152.7 | 37.4 |
| **UTR-6/UTR-6** | 3 | 150.7 | 47.0 |
| **UTR-1/UTR-2** | 12 | 138.2 | 21.5 |
| **UTR-1/UTR-3** | 18 | 145.8 | 26.8 |
| **UTR-1/UTR-4** | 5 | 164.4 | 24.6 |
| **UTR-1/UTR-5** | 4 | 155.6 | 49.3 |
| **UTR-1/UTR-6** | 8 | 145.8 | 12.2 |
| **UTR-2/UTR-3** | 31 | 135.9 | 30.6 |
| **UTR-2/UTR-4** | 7 | 138.4 | 40.3 |
| **UTR-2/UTR-5** | 22 | 130.7 | 31.7 |
| **UTR-2/UTR-6** | 14 | 150.4 | 35.5 |
| **UTR-3/UTR-4** | 3 | 119.7 | 21.8 |
| **UTR-3/UTR-5** | 22 | 144.3 | 33.7 |
| **UTR-3/UTR-6** | 14 | 148 | 24.7 |
| **UTR-4/UTR-5** | 3 | 144.4 | 9.0 |
| **UTR-5/UTR-6** | 12 | 146.9 | 15.5 |
